# The value of connections

**DOI:** 10.7554/eLife.92319

**Published:** 2023-09-19

**Authors:** Vanessa Rossetto Marcelino

**Affiliations:** 1 Melbourne Integrative Genomics, School of Biosciences and Department of Microbiology and Immunology at the Peter Doherty Institute for Infection and Immunity, University of Melbourne Melbourne Australia

**Keywords:** gut microbiome, metabolism, inflammatory bowel disease, gastrointestinal disorders, microbial ecology, Human, Bacteria

## Abstract

High proportions of gut bacteria that produce their own food can be an indicator for poor gut health.

**Related research article** Veseli I, Chen YT, Schechter MS, Vanni C, Fogarty EC, Watson AR, Jabri B, Blekhman R, Willis AD, Yu MK, Fernàndez-Guerra A, Füssel J, Eren AM. 2023. Microbes with higher metabolic independence are enriched in human gut microbiomes under stress. eLife **12**:RP89862. doi: 10.7554/eLife.89862.

The human gut is home to over 30 trillion microbes that form a complex ecosystem (Sender et al., 2016). Each person has a unique and dynamic set of microorganisms in their gut, and researchers have long tried to identify and untangle the reasons for this remarkable variation. The list of factors determining which microbes colonize an individual’s gut is extensive, ranging from diet to contact with pets and farm animals, geographical location, ethnicity, history of medications, and various other individual and lifestyle characteristics (Parizadeh and Arrieta, 2023).

The composition of the gut microbiome has also been linked to a range of health conditions, with loss of species diversity being a common hallmark of disturbed microbiomes (Bidell et al., 2022). These associations have fuelled the idea that the gut microbiome can be used as a non-invasive biomarker of health status, or to improve and maintain human health by introducing beneficial bacteria and removing pathogens from the gut.

However, it is still largely unclear whether changes in the microbiome are the cause or consequence of disease. The challenges in teasing apart the many intricate factors shaping microbiome composition constitute a major roadblock to translating the vast body of microbiome research into clinical practices. Now, in eLife, Iva Veseli (University of Chicago), Jessika Füssel, A. Murat Eren and colleagues report that the extent to which bacteria can synthetize their own food is a significant trait determining the composition of unhealthy gut microbiomes (Veseli et al., 2023).

The team – who are based at various research institutes in the United States, Denmark and Germany – analysed gut microbiomes associated with inflammatory bowel disease (IBD) and other gastrointestinal conditions. The diversity of microbes in these communities is typically low due to antibiotics, diarrhoea and other features linked to a stressed gut environment. Unlike most previous studies that looked at taxonomic or species composition, Veseli et al. investigated the genome content of bacteria, focusing on their capacity to produce and metabolize essential nutrients, such as amino acids, carbohydrates and vitamins.

They found that stressed gut environments contained bacteria whose genomes encoded complete pathways to biosynthesise essential nutrients – i.e., they show high metabolic independence. In contrast, bacterial genomes from healthy individuals contained seemingly incomplete metabolic pathways, suggesting that they rely more extensively on nutrients produced by their peers to survive, also known as cross-feeding (Figure 1).

**Figure 1.**
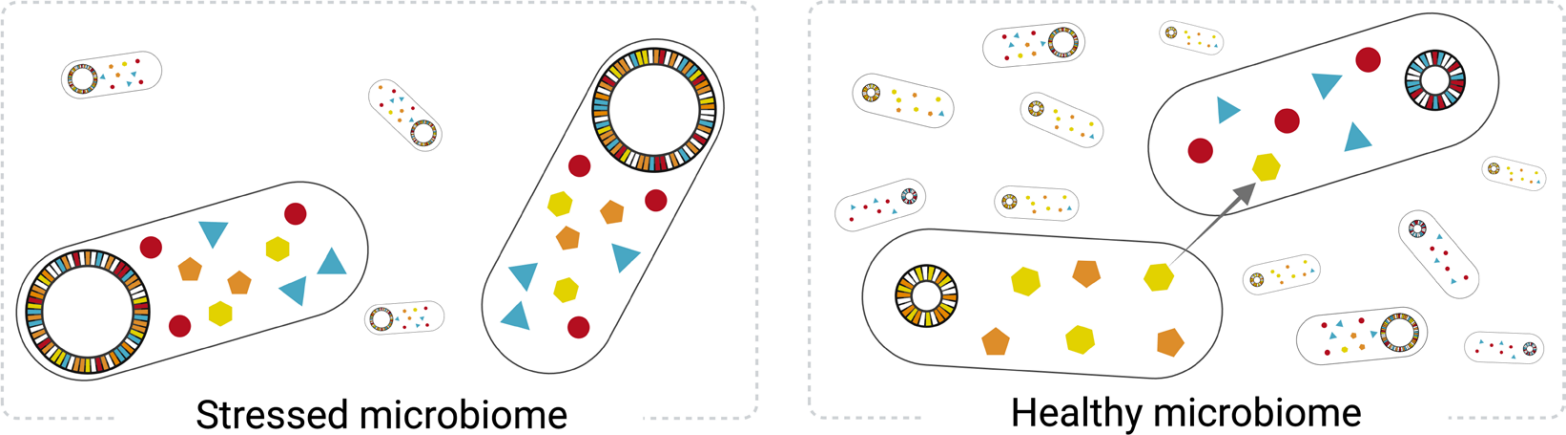
Bacteria living in stressed and healthy gut environments have distinct metabolic potentials. The stressed gut microbiome (left) is predominantly colonized by a low diversity of bacteria whose genomes encode pathways for synthesising a range of essential metabolites, represented by the coloured shapes. These ‘metabolically independent’ bacteria are expected to generate their own food. Conversely, gut microbiomes associated with healthy individuals (right) are enriched in bacteria that seem genetically incapable of synthesising all the nutrients they need, suggesting that they rely more extensively on nutrients produced by other bacteria.

Image credit: Figure created with BioRender.com.

Next, Veseli et al. asked whether the overall metabolic independence of gut bacteria could be used as a biomarker of health status. First, the team developed an open-source software platform to systematically quantify metabolic independence from high-throughput sequencing data. They applied their newly developed approach to over 300 deeply sequenced stool samples from individuals with IBD and healthy controls. They then showed that, with the help of machine learning, it is indeed possible to accurately identify individuals with IBD based entirely on the estimated self-sufficiency of their microbiome.

To expand the scope of their findings beyond IBD, Veseli et al. showed that a short dose of antibiotics taken by healthy volunteers leads to a sharp increase in the proportions of self-sufficient gut bacteria, followed by a gradual recovery of bacteria that seem to rely on cross-feeding. These results support the claim that high metabolic independence is a hallmark of poorly diverse, stressed gut ecosystems, which can be used as a biomarker of gut health status. Since it is based on mechanisms rather than the taxonomic identity of microbiome members, the approach proposed by Veseli et al. is likely to be more robust to the ethnicity, geographic location and lifestyle factors that have obscured associations between microbiomes and health status in the past (Sze and Schloss, 2016; Gaulke and Sharpton, 2018).

The implications of this study bring a new perspective to the microbiome field. Bacteria typically labelled as pathogens for their association with unhealthy microbiomes might not be causative disease agents as previosuly assumed. Instead, they might simply be the only ones capable of surviving in a poorly diverse gut. The study also adds key evidence to the growing awareness of the relationships between microbial cross-feeding and microbiome composition, paving the way to test interesting questions in future research (Wang et al., 2019; Marcelino et al., 2023; Gralka et al., 2020; Watson et al., 2023). For example, what are the roles of bacteria with high metabolic independence in re-establishing a healthy gut microbiome after disruption? If self-sufficient bacteria are at the bottom of the microbial food chain, one can wonder whether these presumed villains will become heroes in restoring the gut ecosystem. These new perspectives bring us one step closer to fully benefit from the diagnostic and therapeutic potential of the human gut microbiome.
